# Primary Care Networks and Eritrean Immigrants’ Experiences with Health Care Professionals in Switzerland: A Qualitative Approach

**DOI:** 10.3390/ijerph16142614

**Published:** 2019-07-23

**Authors:** Carla Wallimann, Andreas Balthasar

**Affiliations:** Department of Health Sciences and Health Policy, University of Lucerne, 6002 Lucerne, Switzerland

**Keywords:** migrant health, primary care, network, health professionals, transcultural competence

## Abstract

Growing migration in European countries has simultaneously increased cultural diversity in health care. Migrants’ equal access to health care systems and migrant friendly health care have therefore become relevant topics. Findings gathered in recent years have mainly focussed on the perspective of care providers, whereas this study includes migrant perspectives. It explores the primary care network of Eritrean immigrants in Switzerland as well as their experiences of interacting with health professionals. Semi-structured face-to-face interviews with intercultural interpreters from Eritrea were conducted. On the basis of a thematic analysis, the study identified the important informal and formal contacts in these Eritrean immigrants’ primary care networks and the specific forms of support each actor provides. In this network, encounters with health professionals were predominately expressed positively. The main barriers reported were language difficulties and intercultural understanding. On the basis of the participants’ statements, six key lessons for practice have been derived. These lessons are specifically important for facilitating Eritrean immigrants’ access to the Swiss health care system. Nevertheless, they are also relevant for other groups of migrants in European countries.

## 1. Introduction

As in many European countries, Switzerland’s immigrant population has been growing in recent years [1,2]. In 2015, Switzerland’s population was 24.6% foreign [2]. As a result of increased migration, cultural diversity in health care has also increased [3]. Migrant friendly health care provision and equal access to health care are topics that have moved into political, social and scientific focus in Switzerland and abroad [3,4,5,6,7,8,9,10,11]. Although Switzerland’s migrant population is heterogeneous, studies have shown that the majority of migrants have a lower level of education and income than the population’s average, while poverty, unemployment and health problems are more prevalent in persons with a migration background [2,12,13]. Socio-economic disadvantages and psychosocial stress factors linked to migration are known to have a negative impact on health [6,14,15,16,17,18].

Access to and interaction with the Swiss health care system are influenced by multiple factors at structural/political, institutional and individual levels [19,20,21]. Most barriers and difficulties are due to the interrelation of social determinants, individual characteristics and institutional conditions [20,21]. Health professionals perceive communication to be the major challenge in encounters with migrants: language difficulties and different intercultural understanding of roles, health and illness, and examination and treatment options [21,22,23]. Other important aspects are a patient’s health literacy [24,25] and knowledge of the health care system and services [19,21,26]. To deal with diversity and to overcome difficulties, intercultural interpreters play an important role [21,22,27,28]. Moreover, fostering health literacy of patients with a migration background and the transcultural competencies of healthcare professionals are emphasised [5,21,22].

Despite the findings gathered in recent years, there are still knowledge gaps regarding the quality of care in relation to communication difficulties, and barriers for migrants in accessing healthcare [4]. Many studies have investigated provider perspectives but research lacks the inclusion of migrant perspectives [13,23,29]. Furthermore, focusing on specific migrant groups would help to clarify cultural differences and experiences of health care [29].

The aims of this qualitative study were to map Eritrean immigrants’ primary care networks and to capture their experiences with health professionals within these networks. By the primary care network, we mean the persons and institutions that the migrants contact first in case of health problems. Answering the above mentioned questions should contribute to better understanding for providers and policy makers, and to fostering satisfactory interactions between migrants and healthcare professionals.

The focus was set on immigrants from Eritrea as they have accounted for the largest group of asylum seekers and recognised refugees in Switzerland for some years [30]. In addition, the cultural background of immigrants from Eritrea is very different from the Swiss context. The focus was set on the role of primary care physicians because in Switzerland, general practitioners are usually the first contact point in case of illness [31], whereas a general practitioner system is not known in Eritrea [26].

## 2. Materials and Methods

The empirical basis of this paper is eight semi-structured face-to-face interviews with intercultural interpreters. Participants eligible for this study were adult immigrants from Eritrea living in Switzerland and being active as intercultural interpreters. Intercultural interpreters were chosen since they are seen as bridge-builders between patients and healthcare professionals. They know both the Eritrean and the Swiss culture, and are able to report on the experiences of different individuals.

In Switzerland, the placement of qualified intercultural interpreters is organised by regional agencies [32]. For the recruitment, an e-mail accompanied by an information sheet was sent to all eleven institutions in German speaking Switzerland, which are organising the placement of translators (there are four different language regions in Switzerland of which the German speaking part is the largest). Seven of them forwarded the request to their Eritrean interpreters. Eight volunteers responded to this request and took part in the study. All participants signed an informed consent form. According to the Ethics Committee Northwest and Central Switzerland, ethical approval was not required for this study as it did not involve research on disease or the structure and functioning of the human body.

The interviews were based on an interview guide with a series of open questions to identify the institutions and the persons relevant for the refugees in case of health issues. The interview guide also included the task of mapping relevant individuals and or institutions in dealing with health issues by using the concept and technique of the “social atom” [33]. This technique, originally developed by Jacob Levy Moreno, is based on a systemic approach and allows visualising relations and their quality. Furthermore, the interview included open questions on experiences with health professionals in Switzerland and on relevant cultural aspects. Each interview took about an hour and was conducted in German. All interviews were audiotaped and transcribed verbatim. In addition, the author made field notes.

Table 1 shows the demographic and work characteristics of the eight intercultural interpreters interviewed. With four female and four male participants, the gender balance was even. The age ranged from 26 years to 53 years with a mean age of 41.1 years. On average, the participants had been living in Switzerland for 13.6 years.

An initial inclusion criterion was that the participants were born in Eritrea. However, one participant was born in Ethiopia, another in Sudan. Nevertheless, they were included as they are familiar with the Eritrean culture and interpret for Eritrean people.

All participants were qualified intercultural interpreters and on average had been active for 5.3 years. As a group, they had a high educational level, with half of them having received a tertiary education. All participants spoke Tigrinya, the most widespread language in Eritrea, plus English and German. In addition, participants covered several other languages spoken in Eritrea. The religious affiliations in the sample were comparable to the distribution among Eritreans in Switzerland, where the vast majority are Orthodox and Catholic [26].

Participants bring in a broad variety of work experiences in diverse health care settings in urban and rural areas in German-speaking Switzerland. Their clients are Eritrean immigrants of all ages. The participants described their clients as people with insufficient local language skills, most of whom have been living in Switzerland for less than five years (sometimes up to 10 years) and with little contact with the local population. Participants reported that the number of interpreting assignments varies, but usually ranges from three to five assignments per week.

After reading and familiarisation with the data, a complete coding across the entire dataset was carried out, after which patterns and themes were identified. The coding and the analysis were based on the approach proposed by Braun and Clarke [34]. As a tool to assist coding and analysis, the computer software MAXQDA 10 (VERBI Software GmbH, Berlin, Germany) was used. Quotations from the interviews were selected to illustrate the findings. All quotations were translated into English. The results were discussed with an Eritrean epidemiologist who lives in Switzerland and studied medicine in Eritrea and his feedback is incorporated into this article (the corresponding parts are declared).

## 3. Results

### 3.1. The Primary Care Networks of Eritrean Immigrants in Switzerland

**Figure 1:**
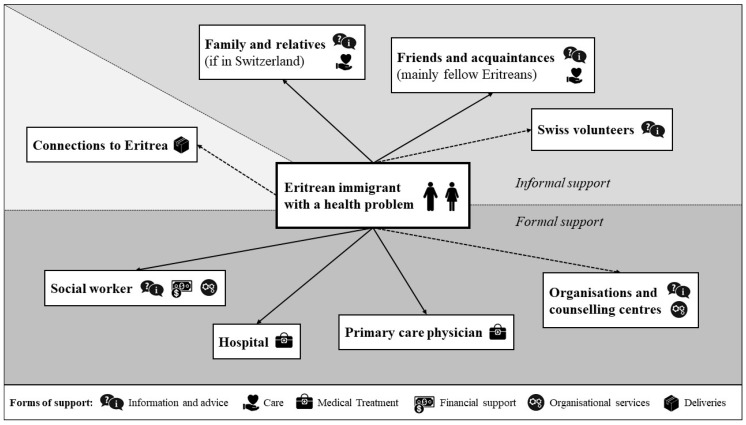
Overview of an Eritrean immigrant’s primary care network (main actors and main support forms). Source: own illustration based upon the interviews.

Participants’ statements about Eritrean immigrants’ primary care networks in Switzerland were very similar. Figure 1 provides an overview of the most important informal and formal points of contact and their main forms of support. The characteristics of each actor are described below, with a particular weight on the primary care physician. Although in the interviews it was emphasised that resident status plays a relevant role in the assignment of a doctor, interpreter or financial support, the pictured network did not differ fundamentally between individuals with different resident statuses. Where applicable, differences are described.

#### 3.1.1. Informal Support

Family and relatives are important for giving advice and care in the case of illness or health related issues. However, many Eritrean immigrants do not have family in Switzerland, and this fact is linked with psychological stress. For those, in particular adolescents and young adults under 25 years, friends and acquaintances or professionals play an essential role.

As a result of language barriers and cultural aspects, these friends and acquaintances are mostly fellow Eritreans (contacts from the asylum centre, language school or Eritreans who have lived in Switzerland longer). They provide care or information on where to seek help.

Volunteers were named as important support, and not only in case of illness. They support integration and can be persons of trust, who are contacted in cases of questions or health problems. Volunteers are mostly Swiss who have been recruited by an NGO such as Caritas, which is engaged in supporting the integration of migrants. In some cases, volunteers act on an individual basis and met the immigrant at a neighbourhood event, for example.

In some cases, the connection to relatives in Eritrea seems relevant, too, for example to import healing clay, plants and herbs to support recovery. However, from the experience of the Eritrean doctor/epidemiologist, immigrants rarely talk about their health problems with relatives in their home country, only if the impairment is chronic and cannot be concealed.

The meaning of religion and religious persons was assessed inconsistently and is not displayed in the figure. The majority opinion was that religious persons are seldom consulted for health problems (though they can sometimes be consulted in cases of mental health problems). However, for some individuals, the use of sacred water, prayer and attendance at holy mass in their mother tongue play a role in staying healthy or in recovery.

#### 3.1.2. Formal Support

Concerning formal support, the role of social workers is particularly stressed. In Switzerland, every person seeking asylum is entitled to assistance from a social worker. Initial support is provided in the federal asylum centres. Once the decision on the asylum seeker’s residence status has been made, support is organised at the cantonal level. Social workers provide information about medical support and the health system in Switzerland, and they are responsible for the financial support to which migrants are entitled. Contact with social workers is described as fundamentally important as they provide relevant information not only concerning health, but also about life in Switzerland and integration. For people in asylum centres and in particular minors, social workers are often the first contact in the case of health problems and they are typically the ones to make referrals to a physician. However, social workers from the social welfare office are also frequently contacted, for example, when it comes to financial issues or for support in organising an appointment or an interpreter.

Eritrean immigrants seek medical treatment in hospital or at their primary care physician, though not typically the pharmacy. In the participants’ opinion, a person chooses to visit a hospital or a primary care physician based on the duration of their stay in Switzerland and resident status, knowledge of and experience with the health care system, and language skills. While people are waiting for the decision on residence status from the State Secretariat for Migration, the primary care physician is assigned by the State. Later migrants can choose a primary care physician freely. Upon arrival in Switzerland, many Eritreans tend to seek medical help at a hospital rather than with a primary care physician, which may be related to the fact that participants reported commonly visiting the hospital in Eritrea, since the general practitioner system is not known there. Another aspect mentioned is that a hospital’s wealth of equipment gives the impression of being superior to any other location and may be a reason for some people to go to hospital. However, when they have learned about and experienced the general practitioner system, the primary care physician may become an important person of trust. The migrants consider it positive that their physician knows them and their health history personally. In Swiss hospitals, the health history of patients who have been previously treated by a primary care physician or in another hospital is often not available, since an e-health database of this information is not very well developed in Switzerland [35]. Another influence on behaviour is some hospitals’ provision of interpreters, whereas an appointment with a primary care physician will usually require the patient to organise (and pay for) an interpreter themselves.

Private or state-run counselling centres and organisations like Caritas or Swiss Red Cross are mentioned as important support sources for information on the health care system and addresses of services and professionals. In some cantons, such organisations coordinate interpreting services or volunteer interpreters. Eritrean immigrants mostly get to know about these options through their social workers, or through relatives and friends.

When people are lacking local language skills, intercultural interpreters are essential support in the interaction between patients and health professionals. Participants stated that Eritrean immigrants frequently face the problem that they do not know contacts for interpreting when health professionals tell them to bring an interpreter with them. A further difficulty is that the financing of interpreters is not fully clear.

### 3.2. Six Key Lessons for Practice

Participants most often cited positive experiences of encounters with health professionals in Switzerland. Physicians and medical staff were described as being nice and helpful. The main barriers reported were language difficulties and intercultural understanding from both sides. On the basis of information from participants, six key lessons for health care professionals in dealing with migrants from Eritrea were derived. In the following, each point is presented and illustrated with examples from the interpreters’ experiences.

#### 3.2.1. The Challenge of Finding One’s Way in a New Health Care System

Participants reported that it not only takes time to get to know the health care system in Switzerland, but also to culturally adapt to it. In addition to information from formal entities and health professionals, information courses in the clients’ native language are viewed as helpful.

When migrants from Eritrea interact with the health system, they face many unfamiliar situations; for example, the need to fix an appointment in advance or examinations and treatments not known in Eritrea (such as gynaecological examinations, school psychological assessments and counselling, occupational therapy, psychomotor therapy or speech therapy). Furthermore, participants noticed that physicians in Switzerland talk quite directly about the severity of a diagnosis and sometimes make predictions, which is often perceived as a challenge. Unfamiliar situations can lead to misunderstandings and to difficulties in building trust with physicians:

Participant: “What decreases trust is if you have to sign. […] To agree to do something. And then all the negative aspects are written there.”

Interviewer: “Ah, the risks before an operation for example?”

Participant: “Yes, the risks are written. […] As an interpreter I experienced, that when they have to sign, they are frightened and do not trust the doctors.”

In another example, a health professional’s query about how the patient rates his/her pain on a scale from 1–10 was addressed:

Participant: “First of all, I need to know if the client understands this scale. Because, mostly they say: What does the doctor mean? What am I supposed to do? Make it five. I am not sure whether they understood this system. [...] Such a scale is easier for a child who grew up here.”

#### 3.2.2. The Relevance of Clear and Sometimes More Detailed Explanations

In order for Eritrean immigrants to understand, learn and cooperate with the system, participants repeatedly stressed that clear explanations about examinations, procedures and treatments are essential. They could all give manifold examples where too few explanations led to misunderstandings and limited cooperation. One example had to do with medication as a treatment for illness: “The doctors often give the drugs just like that. But the people, the refugees, are scared. Why do I need to take this medication [they ask]? [...] It is important to understand how the drug helps you. […] Otherwise they do not take their medicine at home.” While blood testing is one of the most helpful diagnostic methods in Western medicine, blood sampling and testing is often not explained sufficiently before the migrant patient is asked to have blood taken. One interviewee stated: “Blood sampling can be a problem for an Eritrean. They wonder why blood samples were taken. You have to explain that to them. There are simply things done less in their home country but here a lot. And people do not know about it.”

#### 3.2.3. Appropriate Communication to Overcome Language Barriers

As the participants’ clients are people with a lack of local language skills, many of them experience language barriers in their contact with health professionals. A first obstacle occurs in arranging an appointment. Further difficulties exist in explaining their symptoms or understanding instructions they receive. To facilitate adequate communication, participants see three aspects as relevant for the provider. First, the interaction style of a health professional is perceived as influencing the patient–professional interaction significantly (positively or negatively). Participants highlighted the importance of showing interest in a patient’s issues and taking time to build trust and answer questions. One participant expressed: “The clients have language difficulties. That is their weakness. If the doctor is nice, asks questions and answers their questions, then it goes well, the clients ask and tell more.” Secondly, participants emphasised the importance of involving intercultural interpreters to ensure that Eritreans who do not speak German are adequately informed and that the medical procedure is clarified. A third aspect is additional forms that support communication, such as a document with images of the body to be used to explain a health problem or a medical examination or even translated documents. Health care courses in a patient’s native language are seen as valuable to enhance understanding, decision-making abilities and self-confidence. For example, a participant spoke about a client who attended a birth preparation course offered in Tigrinya: “Afterwards she was a self-confident woman from one day to the next and understood everything.”

#### 3.2.4. Cultural Differences Regarding Gender

The interviews showed that the importance of gender for migrants is often underestimated by medical staff. The gender of a physician, medical staff or an interpreter may have a fundamental influence on Eritrean immigrants’ well-being and behaviour in a health care treatment situation. Participants see it as favourable that patients are treated by a person of the same sex. Especially in the field of gynaecology, it is of particular relevance that the physician is female and the interpreter likewise. According to the participants, many Eritreans are not used to medical examinations and have experienced abuse. That makes it difficult for them to evaluate what is going on and to be relaxed. A participant stated: “Yes, that [gender] is important because physicians need to touch the body for example, where they have pain or they want to press and feel the breasts. This is a strange situation for women.” In addition, there are sensitive health issues that individuals do not want to discuss with someone of the opposite sex.

There are a few special features in health care for women. Participants addressed that in gynaecology and obstetrics, careful handling and education is extremely important. Most women did not get sexual education and preliminary gynaecological examinations are not common in Eritrea: “There are no such examinations in Eritrea. Well, there are some when you are sick, but no check-ups. When a woman has such an examination for the first time, it is very, very difficult for her.” An Eritrean doctor/epidemiologist claimed that there has been progress in sexual education in Eritrea, especially in urban schools. In the case of a pregnancy, participants see it as essential to give all-important information about the course of pregnancy and the examinations at the beginning, if necessary with an intercultural interpreter. Birth preparation courses in a women’s mother tongue are also viewed as very valuable. Furthermore, female genital mutilation/cutting is widespread in Eritrea. According to participants, the form and prevalence varies depending on the region of origin, ethnic origin and education. The participants expect physicians to address this sensitive issue and to look carefully to prevent complications.

#### 3.2.5. Mental Health as a Taboo Topic

Participants expressed that many Eritreans were abused or experienced other traumatic events during their migration. In Switzerland, they are confronted with further psychological burdens such as the lack of a family network, unclear residence status and the lack of occupation. In our participants’ opinions, many Eritrean immigrants needed support to process these experiences. Physical discomfort is usually discussed openly. In contrast, mental health is commonly a taboo subject and associated with prejudices: “When you say that you are stressed or depressed, people judge you to be crazy, you know. That is why they do not want to talk freely about it.” As a result of taboo, stigmatisation and a lack of knowledge among Eritrean immigrants about mental health issues and treatment options, they often reject psychological health care. In psychiatry, an additional effort is needed to build a foundation for treatment.

#### 3.2.6. Differences in Individual Situations and Cultural Backgrounds among People from the Same Country

Our participants’ had the impression that health care professionals often have too little information about an Eritrean immigrants’ cultural and social background. Participants highlighted that in Eritrea, there are nine different ethnic groups with their own languages and cultures. Moreover, migration to Switzerland often takes years and shapes the culture and behaviours of immigrants. Experiences in Switzerland also have an influence.

Language skills were considered an important factor for integration and navigation in the health care system. However, psychological stress rooted in Eritrean immigrants’ experiences and life situations is viewed as a potential obstacle to learning the local language and is thus hampering the integration process. A participant expressed his opinion in the following words: “They came to Switzerland through a life-threatening odyssey and are traumatized. At school they have no concentration, so six months are over without being able to speak German. […] Many Eritreans are physically here in Switzerland, but mentally they are somewhere else. […] Many have posttraumatic stress disorder. […] These people need support.”

## 4. Discussion

Describing and analysing communication in clinical reality has been the focus of many anthropologic and cross-cultural studies [36,37]. Clinical reality is understood as the result of a construction process in the exchange of a patient and medical doctor [38]. This paper contributes to this discussion with a special focus on Eritrean immigrants in Switzerland. On the basis of the rich statements of the intercultural interpreters, the study is able to show what Eritrean immigrants’ primary care networks in Switzerland look like, how they experience interaction with health professionals in this network and what they consider to be important in the encounters.

Important points of contact within the primary care network include informal (family, friends, volunteers) and formal contacts (social workers, primary care physicians, hospitals, organisations and counselling centres) in Switzerland, as well as connections to Eritrea. Each contact is characterised by particular forms of support, such as information provision and advice, care, medical treatment, financial support, organisational services, or deliveries. The description of the network also shows the barriers to finding and getting informal and formal support. According to the participants, obstacles are mainly attributed to the lack of one of the following: a family network, knowledge of the Swiss health care system and culture, German language skills and the financing of intercultural interpreters. These aspects also influence whether migrants from Eritrea seek medical treatment in a hospital or at a primary care physician’s office. These findings are in line with international research reporting that immigrants often struggle with the unfamiliarity of the health care system in the host country and experience language barriers to access health care [19,20,23,26,29]. Participants agree with research and official institutions seeking to both promote immigrants’ Swiss health competence [14,21,39] and improve the conditions for of intercultural interpreters [14,21,22,27].

The interviews also provide a wide range of information about the experiences of Eritrean immigrants with health professionals and about cultural and social dynamics. It is pleasing that the participants reported many positive experiences of encounters with health professionals in Switzerland. However, they all reported barriers, mainly rooted in the lack of German language and intercultural understanding. This goes along with findings of research of health care providers’ perspectives that reported difficulties in interaction [21,22,23]. Participants expressed the clear wish to improve and requested ideas for how to deal with these barriers and with cultural differences.

On the basis of the data gathered, six lessons for practice were derived: the challenge of finding one’s way in a new health care system; the necessity of clear and sometimes detailed explanations; the role of appropriate communication to overcome language barriers; cultural differences between genders and their roles; mental health as a taboo topic; differences in individual situations and cultural backgrounds among people from the same country. These key lessons address important aspects of transcultural competence of health staff, which is currently a widely discussed topic in assuring quality health care for migrants [3,5]. Transcultural competence means the general ability to grasp and understand the individual life context in a particular situation and to derive appropriate, adapted ways of acting [40,41]. This requires empathy, openness, interest, self-reflection and knowledge of the individual’s environment, both in his or her country of origin and in his or her current situation [3,40,42]. Furthermore, it is important to recognise that cultures are subject to change and that every individual has both a personal culture and shared values and attitudes of their sociocultural group(s) [43]. Each key lesson can apply to other migrant groups with regard to attitude, while the content can differ between cultures.

The present study provides specific information for health care providers about the experiences and needs of Eritrean immigrant patients in the Swiss health care sector. The present study does not discuss all aspects and details mentioned by the participants, and other sources are insightful regarding various different aspects of immigrants’ experiences. This study may therefore be complementary to, for example, the study of Eyer and Schweizer [26] on the Eritrean diaspora in Switzerland that provides broad cultural information on Eritrean immigrants in different areas of life. Asefaw [44], as another example, deals in detail with female genital mutilation/cutting in Eritrean women.

Regarding the sample, some characteristics must be taken into account. The study is limited to the information of eight volunteers. This clearly restricts the meaningfulness of the results. However, by the fact that at the end of the study, similar and complementing topics and statements arose, we have the impression of having reached a good level of valid and saturated information. Most of the participating interpreters had a high level of education. Besides their work as intercultural interpreters, some of them had experience in health or social care. According to the participants, many of their Eritrean colleagues would be afraid to take part in such a study because they are not familiar with research and have trust issues due to past experiences in Eritrea or during their migration [45,46]. These aspects may also have caused the results to be bias. To counteract the limitations of the methodical approach, the findings were discussed with an Eritrean epidemiologist who lives in Switzerland. His feedback is also stated in the article. Nevertheless, and precisely because of language and issues of trust, the method of qualitative interviews with intercultural interpreters is regarded as efficient. The intercultural interpreters brought in different perspectives, whether as an immigrant and patient themselves, or based on the experiences of and with their clients. This enables a broad collection of experiences and perspectives from which we can learn. The method is considered suitable for research on other migrant groups or to investigate specific health issues, since participation can be gained through key persons of trust.

## 5. Conclusions

This study identified the relevant informal and formal contacts in the primary care networks of Eritrean immigrants in Switzerland. Through the eyes of intercultural interpreters, it gives insight into Eritrean immigrants’ experiences in interactions with health professionals in this network. The barriers they perceived are similar to those health professionals reported in previous national and international studies. The key lesson provides specific information on immigrants from Eritrea but it also has some practical implications for the quality of care for migrants in general. This knowledge contributes to making the health system accessible and to meeting immigrants’ needs. The multi-perspective method can be considered efficient in its use of intercultural interpreters and can therefore be recommended for research with other migrant groups. However, the specific role and characteristics of the group of interpreters must be taken into account.

## Figures and Tables

**Table 1:** Demographic and work characteristics of the participants.

Demographic Characteristics	N = 8
Gender (*n*)	Female (4), male (4)
Age in years (mean)	26–53 (41.1)
Place of birth (*n*)	Eritrea (6), Ethiopia (1), Sudan (1)
Duration of stay in Switzerland in years (mean)	6–33 (13.6)
Duration of activity as an intercultural interpreter in years (mean)	1–8 (5.3)
Languages spoken (*n*)	Covered by all: Tigrinya (8), English (8), German (8) Further: Amharic (6), Tigre (2), Bilen (1), Arabic (1), French (1), Italian (1)
Educational background (*n*) (education was acquired in Eritrea or in Switzerland. Some participants had completed several studies or training courses)	Tertiary education in social, health, natural or environmental sciences (4), nursing (2), education in the fields of clothing and beauty (2), secondary school (1)
Religious affiliation (*n*)	Orthodox (4), Catholic (2), Protestant (1), non-denominational (1)
**Work Characteristics**	
Geographical area of activity	Urban and rural areas in German-speaking Switzerland
Main fields of activity in health care	Hospitals, in particular maternity wards, surgical wards, mental health facilities, and with primary care physicians
Characteristics of their clients	Eritrean immigrants of all ages with a lack of local language skills, most of them living in Switzerland for less than five years (sometimes up to 10 years)
Frequency of interpreting	About 3–5 assignments per week
